# LncRNA ITGB2-AS1 Could Promote the Migration and Invasion of Breast Cancer Cells through Up-Regulating ITGB2

**DOI:** 10.3390/ijms19071866

**Published:** 2018-06-25

**Authors:** Mengyao Liu, Liyao Gou, Jing Xia, Qun Wan, Yayun Jiang, Shilei Sun, Min Tang, Tongchuan He, Yan Zhang

**Affiliations:** 1 Key Laboratory of Diagnostic Medicine Designated by the Chinese Ministry of Education, Chongqing Medical University, Chongqing 400000, China; 2015111060@stu.cqmu.edu.cn (M.L.); 2015111059@stu.cqmu.edu.cn (L.G.); 2015111061@stu.cqmu.edu.cn (J.X.); 2016111006@stu.cqmu.edu.cn (Q.W.); summer101004@gmail.com (Y.J.); 2017111032@stu.cqmu.edu.cn (S.S.); 2017111031@stu.cqmu.edu.cn (M.T.); 2 Molecular Oncology Laboratory, Department of Surgery, University of Chicago Medical Center, Chicago, IL 60637, USA; tche@bsd.uchicago.edu

**Keywords:** lncRNA, ITGB2-AS1, ITGB2, breast cancer, migration, invasion

## Abstract

In the previous study, we screened a novel lncRNA-ITGB2-AS1, which was down-regulated by bone morphogenetic protein 9 (BMP9) in breast cancer cell. Studying ITGB2-AS1 will lay the foundation for the exploring mechanism of the BMP9 inhibitory effect on breast cancer. The expression analysis related to ITGB2-AS1 in clinical samples was conducted on online websites. The overexpression plasmid or siRNA fragment was transfected into breast cancer cells to alter its gene expression. The MTT assay and flow cytometry were used to measure cell viability and cell cycle. Additionally, cell migration and invasion were detected by wound healing and transwell assay. The results of biological function experiments showed that ITGB2-AS1 could promote the migration and invasion of breast cancer. Furthermore, ITGB2-AS1 increased the mRNA and protein expression of ITGB2. Consistent with ITGB2-AS1, ITGB2 exerted the promotion effect on the migration and invasion of breast cancer and activated integrin-related FAK signaling. The OL plasmid expressing the truncation of ITGB2-AS1, which was complementary to ITGB2, was essential for activation of FAK signaling. In conclusion, LncRNA ITGB2-AS1 could promote the migration and invasion of breast cancer cells by up-regulating ITGB2.

## 1. Introduction

Cancer is the second leading cause of death after cardiovascular diseases [1], and the incidence of cancer has steadily increased over the years. Breast cancer is the most common malignant disease in women all over the world [2,3]. Although the incidence in China, the largest developing country, was not as high as other high-resource countries historically [4], the mortality of breast cancer remained higher level [5] because of poor diagnostic rates, lack of mammography screening programme [6], and treatment options being unavailable. All these factors may contribute to cancer metastasis and a lower 5-year survival. Emerging evidence implicates inflammation and obesity in cancer progression and distant metastasis [7,8]. However, there is a long way to go to complete our understanding of breast cancer due to the heterogeneity of tumor patients. According to the expression of its estrogen, progesterone, and Her2 receptor, breast cancer can be divided into four subgroups: luminal A-ER positive, PR positive, and Her2 negative; luminal B-ER positive, PR positive, and Her2 positive; Her 2 phenotype-ER negative, PR negative, and Her2 positive; and triple negative phenotype-ER negative, PR negative, and Her2 negative. Recent developments in personalized medicine and immunotherapy [9] have increased the recovery and survival rate to some extent, but we still need to spend a long time in searching for more effective treatment or medicine to cure breast cancer totally.

Bone morphogenetic protein 9 (BMP9), also named growth differentiation factor 2, is a member of the transforming growth factor-β subfamily [10]. It has been confirmed that BMP9, a potent osteogenetic factor, plays various roles in cancers. For example, BMP9 can inhibit the growth of lung adenocarcinoma [11], colon cancer [12], and osteocarcinoma [13], but exert a promotion effect on the proliferation and migration of liver cancer cell [14]. Additionally, in our previous research, we have found that BMP9 can inhibit the proliferation and migration of the breast cancer cell lines MDA-MB-231 [15] and SK-BR-3 [16]. However, the mechanism concerning the inhibition effect of BMP9 on breast cancer cells is not thoroughly understood. LncRNA (long, non-coding RNA), a class of RNA with a length greater than 200nt that does not have a protein coding function, is reported to regulate gene expression in diverse ways [17,18]. To delineate the mechanism of BMP9 on the breast from the perspective of lncRNA, we compared the differences in lncRNA expression between MDA-MB-231 cell transfected with BMP9 and GFP adenovirus. After screening with the gene chip and further validation with quantitative PCR, it has been proved that BMP9 could decrease the expression of ITGB2-AS1 significantly, so ITGB2-AS1 was chosen as research subject. In this article, we firstly measured the effect of ITGB2-AS1 on breast cancer cell in vitro. In terms of mechanism, the relationship between ITGB2-AS1 and ITGB2 was determined by database analyses and cell experiments. Because the FAK signaling is the first pathway activated by the integrin family, we measured the phosphorylation of FAK protein. The aim of this paper is to elucidate the influence of lncRNA ITGB2-AS1 on breast cancer firstly, which will lay the foundation for studying the mechanism of the BMP9 inhibitory effect on breast cancer in the future.

## 2. Results

### 2.1. The Expression Analysis of ITGB2-AS1 on Online Database

**Figure 1:**
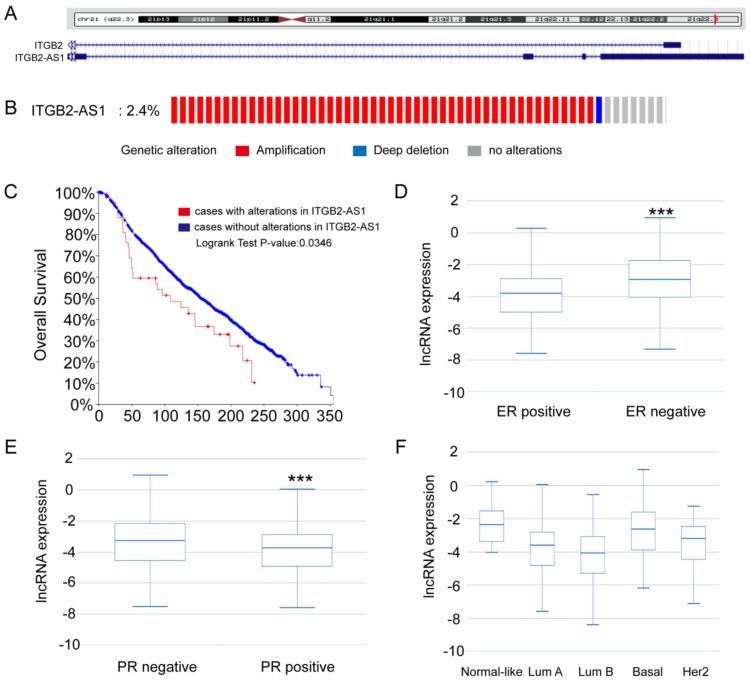
The expression analysis of ITGB2-AS1 on online database. (**A**) The gene location in UCSC genome website; (**B**)The proportion of genetic alteration in METABRIC sub-database on cBioPortal website; (**C**) The overall survival curve of METABRIC sub-database on cBioPortal website; (**D**) The expression of ITGB2-AS1 in ER-positive and ER-negative breast cancer samples of TANRIC database; (**E**) The expression of ITGB2-AS1 in PR-positive and PR-negative breast cancer samples of TANRIC database; (**F**) The expression of ITGB2-AS1 in five pathological types of breast cancer in TANRIC database. *** *p* < 0.001.

From the UCSC genome browser, it can be shown that ITGB2-AS1 was located in complementary strand to ITGB2 gene on chromosome 21. However, there was no thorough paper about ITGB2-AS1, except that ITGB2-AS1 was reported to have high co-expression with multiple cancer genes (IKZF1, LCK, and WAS) [19]. To identify the relationship between ITGB2-AS1 and breast cancer, we first analyzed the expression difference of ITGB2-AS1 in breast cancer clinical specimen database. On the one hand, 2.4% of 2059 samples had a higher expression of ITGB2-AS1 in METABRIC breast cancer sub-database on cBioPortal online website [20,21,22], and higher expression group had a poorer overall survival rate (*p* < 0.05). On the other hand, the expression of ITGB2-AS1 in ER, PR negative samples was higher significantly than that in ER, PR-positive samples in TCGA-BRCA sub-database [23,24] in TANRIC online website (Figure 1D,E). Additionally, there were different expressions of ITGB2-AS1 in five pathological types of breast cancer shown in Figure 1F (*p* < 0.001). Taken together, these data suggested that ITGB2-AS1 may accelerate the progression of breast cancer.

### 2.2. Overexpression of ITGB2-AS1 Could Promote the Migration and Invasion of MCF-7 Cell

**Figure 2:**
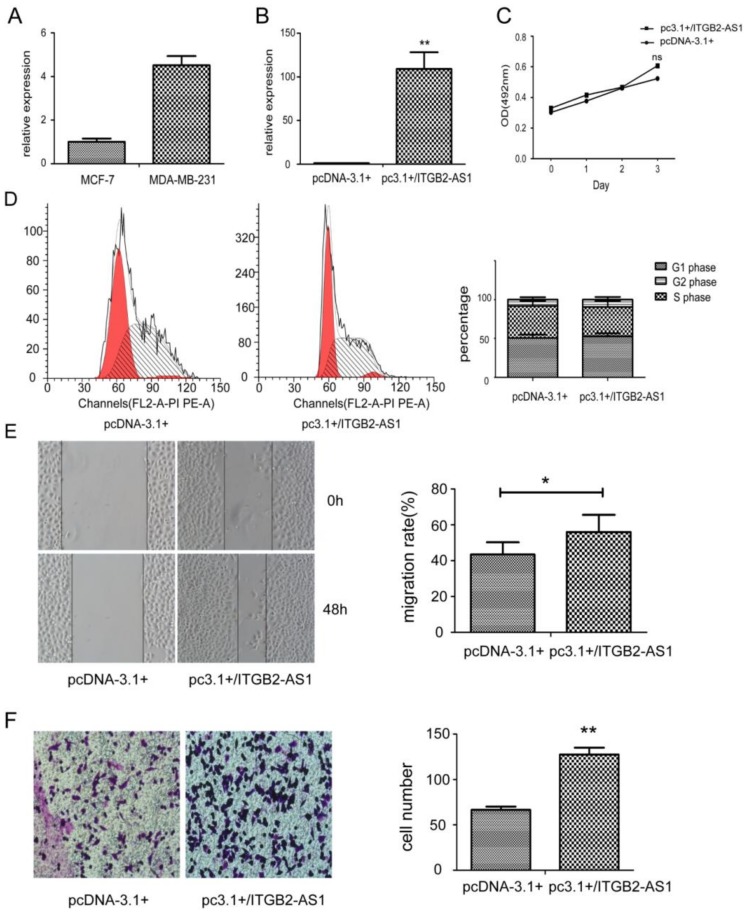
Overexpression of ITGB2-AS1 could promote the migration and invasion of MCF-7 cell. (**A**) The expression of ITGB2-AS1 in MDA-MB-231 and MCF-7 was measured by Q-PCR; (**B**) The validation of overexpression plasmid in MCF-7 by Q-PCR; (**C**) MTT assay; (**D**) The cell cycle was measured by flow cytometry. Left panel: X-axis: cell number; Y-axis: DNA content; (**E**) The migration was evaluated by wound healing assay; (**F**) The invasion was detected by transwell assay and stained by crystal violet (400×). Data are shown as mean ± SD. * *p* < 0.05, ** *p* < 0.01 vs. control groups.

Due to the expression differences between MCF-7 and MDA-MB-231 cell (Figure 2A), we firstly transfected overexpression plasmid of ITGB2-AS1 into MCF-7 cell. The transfection efficiency was verified in Figure 2B by Q-PCR (quantitative PCR). From the data of MTT assay in Figure 2C, it is apparent that there was no significant differences in proliferation after plasmid transfection. Unsurprisingly, result of flow cytometry in Figure 2D showed no significant distinction of cell cycle compared to control and overexpression group. However, the migration rate of overexpression group measured by wound healing assay was higher than control group (Figure 2E). In transwell assay, there were more invasive cells of overexpression group than control group. These data showed that higher expression of ITGB2-AS1 will enhance cell migration and invasion in breast cancer.

### 2.3. Knock-Down of ITGB2-AS1 Exerted Inhibitory Effect on Breast Cancer

**Figure 3:**
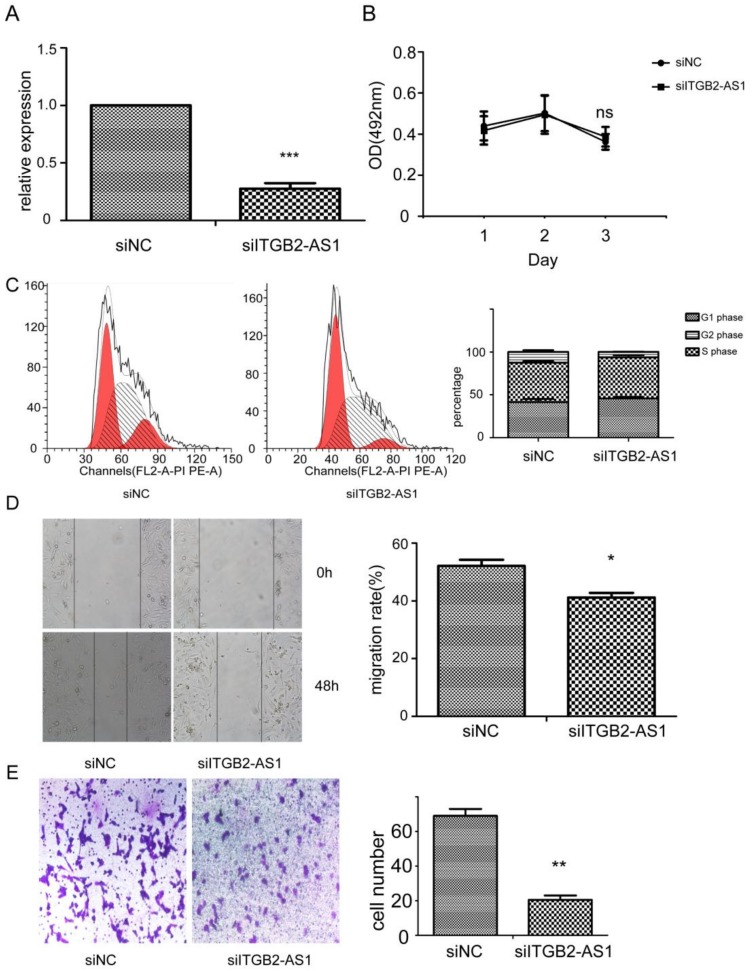
Knock-down of ITGB2-AS1 will exert inhibitory effect on migration and invasion of breast cancer cell. (**A**) The efficacy of siRNA fragment was verified by quantitative PCR; (**B**) The cell viability was detected by MTT assay; (**C**) The cell cycle distribution was measured by flow cytometry. Left panel: X-axis: cell number; Y-axis: DNA content; (**D**) The migration was evaluated by wound healing assay; (**E**) The invasion was detected by transwell chamber assay. Stained with 0.1% crystal violet (400×). Data are shown as mean ± SD. * *p* < 0.05, ** *p* < 0.01, *** *p* < 0.001 vs. control groups.

Next, we transfected the siRNA against ITGB2-AS1 to knock-down its expression in MDA-MB-231 cell. Figure 3A reveals that the siRNA successfully decrease the expression of ITGB2-AS1. Figure 3B,C shows that ITGB2-AS1 had no effect on cell viability and cycle. However, the migration rate of siITGB2-AS1 group was significantly less than siNC group, confirming that ITGB2-AS1 can enhance cell migration. In accordance with previous research, the invasive cell number of siITGB2-AS1 group was also less than siNC group. In summary, all these data indicated that ITGB2-AS1 exerted promotion effect on migration and invasion of breast cancer cell again from the opposite perspective.

### 2.4. There Is a Positive Correlation between the Expression of ITGB2-AS1 and ITGB2

**Figure 4:**
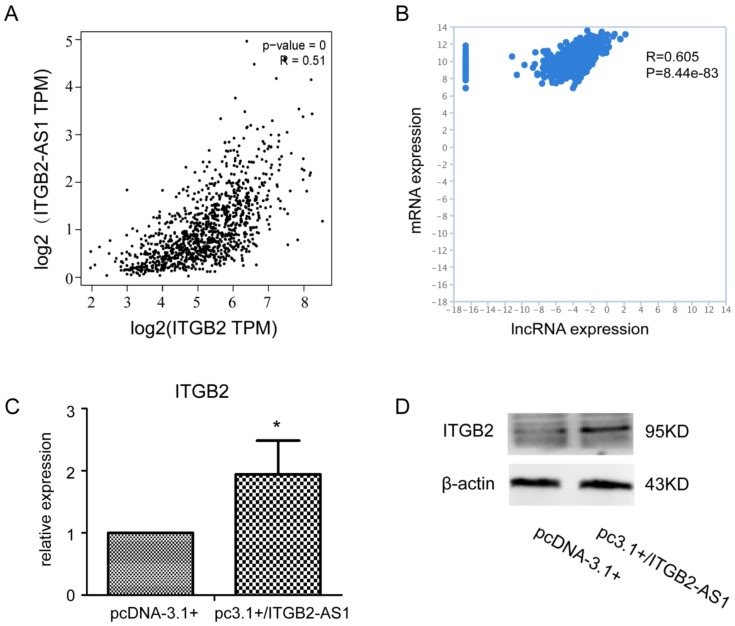
There is a positive correlation between the expression of ITGB2-AS1 and ITGB2. (**A**) The correlation between ITGB2-AS1 and ITGB2 in GEPIA website; (**B**) The correlation between ITGB2-AS1 and ITGB2 in TANRIC website; (**C**) Q-PCR assay in MCF-7 cell; the β-actin was used as internal reference; (**D**) The ITGB2 protein expression was determined by western blotting in MCF-7 cell. * *p* < 0.05 vs. control group.

To further investigate the mechanism of this promotion effect, we first studied the relationship between ITGB2-AS1 and ITGB2 in view of its special position on chromosome. As shown in Figure 4A (BRCA sub-database in GEPIA website,) [25,26] and Figure 4B (TCGA-BRCA sub-database in TANRIC website [22]), their expression in clinical samples had a positive correlation. The result of PCR and Western blotting revealed that overexpression of ITGB2-AS1 can increase the mRNA and protein level of ITGB2 in MCF-7 cell. Taken together, these results provide important insights into mechanism under promotion effect of ITGB2-AS1.

### 2.5. Overexpression of ITGB2 also Could Facilitate the Migration and Invasion of MCF-7 Cell

**Figure 5:**
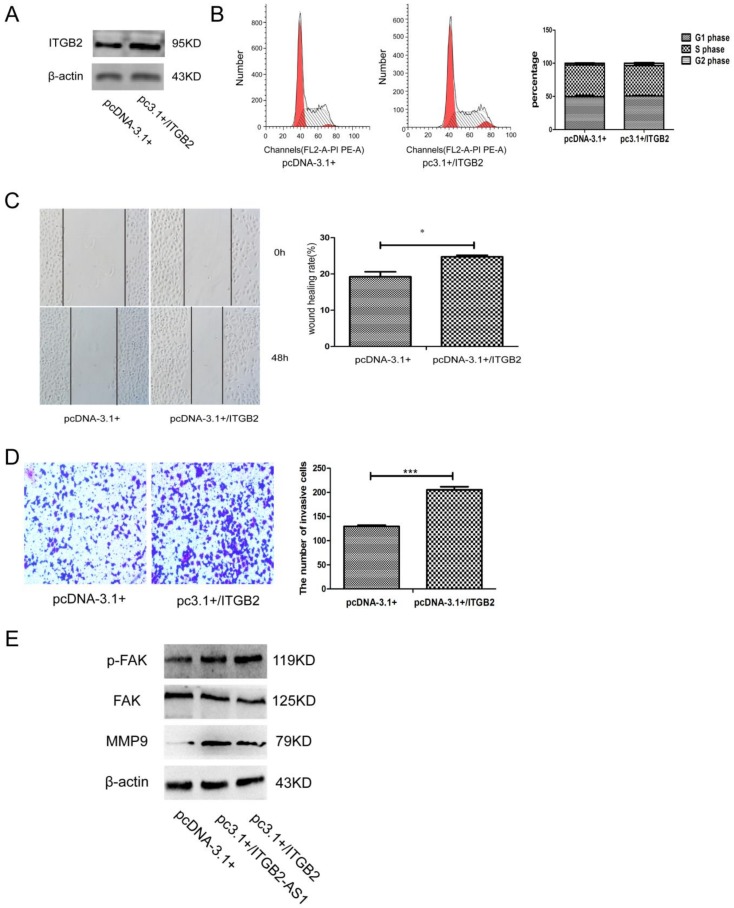
Overexpression of ITGB2 also could facilitate the migration and invasion of MCF-7 cell. (**A**) The verification of ITGB2 plasmid with western blotting assay; the β-actin was used as internal reference; (**B**) The cell cycle was evaluated by flow cytometry. Left panel: X-axis: cell number; Y-axis: DNA content; (**C**)The migration was detected by wound healing assay; (**D**) The cell invasion measured by transwell chamber assay, stained by 0.1% crystal violet (400×); (**E**) The detection of FAK signaling and MMP9 protein by western blotting assay. * *p* < 0.05, *** *p* < 0.001 vs. control groups.

It has been reported that ITGB2 belongs to integrin family, which was closely related to various tumor growth and metastasis. However, the previous research of ITGB2 focused on its effect in leukocytes. To evaluate its effect on breast cancer cell, we constructed ITGB2 plasmid to transfect MCF-7 cell. As can be seen from the data in Figure 5A, the protein expression level was increased significantly after plasmid transfection. Although ITGB2 did not affect cell cycle distribution (Figure 5B); it can enhance cell migration and invasion measured by wound healing and transwell assay (Figure 5C,D). In addition, ITGB2-AS1 overexpression could activate the ITGB2-related FAK signaling and increase the expression of MMP9, a protein related to invasion. Thus, these results show that ITGB2-AS1 could promote the migration and invasion of breast cancer cell by up-regulating ITGB2.

### 2.6. The OL Fragment Plays a Critical Role in the Promotion Effect of ITGB2-AS1 on MCF-7 Cell

**Figure 6:**
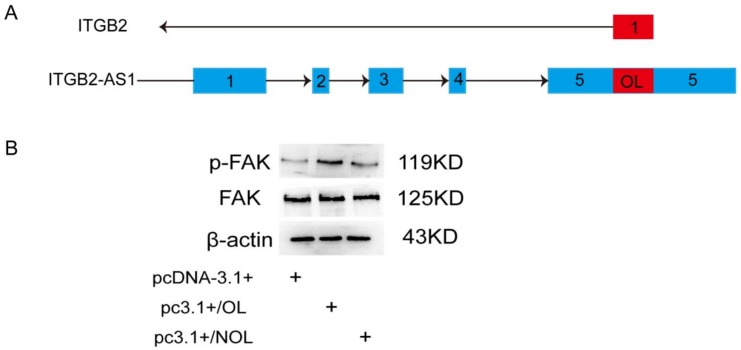
The OL fragment plays a critical role in the promotion effect of ITGB2-AS1 on MCF-7 cell. (**A**) The illustration of OL and NOL plasmid. Blue square: exon; arrow: transcription direction. (**B**) The FAK signaling was detected by western blotting.

As shown in UCSC genome browser, there are 231 bases in the ITGB2-AS1 sequence paired with ITGB2 gene according to principle of complementary base pairing. Accordingly, we constructed segmented plasmid of ITGB2-AS1 to detect the effect of this complementary sequence. From the illustration in Figure 6A, the plasmid containing only complementary sequences was named as pc3.1+/OL, and plasmid containing the rest of the sequence was named as pc3.1+/NOL. The result of western blotting suggested pc3.1+/OL plasmid could activate FAK signaling effectively (Figure 6B). These findings indicated that OL fragment plays an important role in promotion effect of ITGB2-AS1 on breast cancer cell.

## 3. Discussion

Patients with advanced breast cancer often have distant organ metastases, such as bone and lung [27]. Additionally, breast cancer patients with bone metastases are always accompanied by osteolytic lesions, which usually cause bone fractures. Due to its osteogenesis, BMP9 has a promising future as a drug for bone fractures. Additionally, in our previous study, BMP9 could inhibit proliferation and migration of breast cancer in bone microenvironment, which provides a clue for combination medication for advanced breast cancer patients accompanied by bone fracture. Therefore, research about inhibitory effect of BMP9 on breast cancer has important clinical significance.

As is well-known, although lncRNA does not encode protein, it can regulate protein expression in various ways. For example, H19 can act as transcription precursor of miRNA [28] or directly bind to miRNA [29]. NKILA can form a ternary complex with the NF-κB/IκBα complex by direct binding to the P65 subunit, thereby affecting the phosphorylation of the NF-κB-related pathway and its mediating breast cancer metastasis [17]. Overexpression of HOTAIR in breast cancer cell lines promotes its proliferation, migration, and invasion [30]. High-expressed UCA1 can recruit more cytoplasmic hn-RNP1 (heterogenous nuclear ribonucleoprotein I) to bind with it, thus competitively inhibiting the binding between hn-RNP1 and p27 mRNA, which will result in a decrease in the expression level of p27 protein and the blocking of the cell in G1 phase [31]. Even one lncRNA can regulate the expression of multiple proteins in a variety of ways. Therefore, it is a wise choice to elucidate BMP9 inhibitory effect on breast cancer from the view of lncRNA.

In our previous research, it has been screened that BMP9 could decrease the expression of ITGB2-AS1 significantly in MDA-MB-231 and MCF-7 cell line [32]. However, it is still unknown if ITGB2-AS1 can alter breast cancer development and progress. To predict its function, we analyzed the expression of ITGB2-AS1 in clinical samples on online website. The result suggested that ITGB2-AS1 may enhance breast cancer development.

Next we verified our prediction in vitro. After transfection with plasmid or siRNA, there was no alteration in cell viability and cell cycle. However, ITGB2-AS1 had promotion effect on migration and invasion of breast cancer, which means it may accelerate cancer progress. On one hand, it has been reported that antisense lncRNA could regulate protein expression of complementary gene [33,34,35]. On the other hand, the phenotype ITGB2-AS1 behaved similarly to the integrin family. So we studied the relationship between ITGB2-AS1 and ITGB2 to investigate its underlying mechanism. There was positive correlation between ITGB2-AS1 and ITGB2 in breast cancer database. Additionally, ITGB2-AS1 could increase ITGB2 protein level in MCF-7 cell. On the basis of proof that ITGB2 facilitated cell migration and invasion, it had been confirmed that ITGB2-AS1, as well as ITGB2, could activate phosphorylation of FAK protein and increase secretion of MMP9. In addition, the turncation of ITGB2-AS1 that is complementary to ITGB2 plays a critical role in activating FAK signaling. The reason why ITGB2-AS1 has no effect on cell proliferation may be that the integrin family directly affects cell movement by interacting with the extracellular matrix (ECM). Therefore, the effect on cell proliferation may be too slight to detect obvious difference in vitro experiment.

In summary, all these data show that ITGB2-AS1 could promote cell migration and invasion through up-regulating ITGB2. However, above results have not been confirmed in vivo so far. Accordingly, the next plan is confirmation of these results in animals and delineating the relationship between BMP9 and ITGB2-AS1.

## 4. Materials and Methods

### 4.1. Cell Line and Cell Culture

Human breast cell line MDA-MB-231 and MCF-7 were purchased from American Type Culture Collection (ATCC) and cultured in Dulbecco’s modified Eagle’s medium (DMEM) supplemented with 10% fetal bovine serum (FBS) and 1% penicillin/streptomycin. All cells were cultured at 37 °C incubator under humidified atmosphere with 5% CO_2_.

### 4.2. Online Database

cBioPortal website: select studies—breast cancer (METABRIC, Nature 2012 & Nat. Commun. 2016); enter genes—ITGB2-AS1; for the remaining parameters, keep the initial settings, then click on “submit query”.

TANRIC website: select “my lncRNA” district; select a cancer type: TCGA breast invasive carcinoma (BRCA); query annotation—input ITGB2-AS1, then click on “submit” button.

### 4.3. RNA Isolation

Cells were treated with corresponding plasmid or siRNA for 48 h. Total RNA was isolated with TRIzol reagent (Invitrogen, Carlsbad, CA, USA) according to manufacturer’s protocol. 1.0 μg RNA was used for cDNA synthesis by reverse transcriptase PCR. The cDNA was detected by a real-time polymerase chain reaction (qPCR) system (Bio-Rad Laboratories, Hercules, CA, USA) using SYBR-Green PCR Master Mix (TaKaRa Co., Dalian, China). After pre-denaturation at 94 for 30 s, the reaction was cycled 40 times in the following way: denaturation at 94 °C for 10 s, annealing at 59 °C for 20 s, and extension at 72 °C for 20 s. β-actin was used as the endogenous control, and sequences of specific primer for ITGB2-AS1 ITGB2 and β-actin were shown in Table 1.

### 4.4. Plasmid Transfection

The plasmids for pcDNA-3.1+,pc3.1+/ITGB2-AS1, pc3.1+/OL, and pc3.1+/NOL were purchased and sequenced in Genscript Co., Ltd. (Nanjing, China). The cDNA for ITGB2 fragment was integrated into pcDNA-3.1+ vector and verified by sequencing in Genescript. The plasmids were transfected into MCF-7 cell using Lipotectime 2000 according to manufacturer’s instruction.

### 4.5. siRNA Transfection

The siRNA pool against ITGB2-AS1 and negative control (NC) was synthesized from Shanghai GenePharma, Co., Ltd. (Shanghai, China). The sequences were shown in Table 2. The MDA-MB-231 cell was seeded into 6-well plate overnight and then transfected siRNA duplexes with R4000 reagent according to manufacturer’s instructions.

### 4.6. Western Blotting

The total protein was isolated with RIPA lysis (Beyotime Institute of Biotechnology, Shanghai, China) according to manufacturer’s protocol 200 µg protein of each group was loaded in SDS-PAGE polyacrylamide gels and then transferred onto PVDF membranes. The PVDF membranes were blocked with 5% bovine serum albumin (Beijing Solarbio Science and Technology, Co., Ltd., Beijing, China) in TBST for 2 h at 37 °C. Then, the membranes were incubated with corresponding primary antibodies overnight at 4 °C. The following primary antibodies were used in the present study: monoclonal rabbit: anti-MMP9, anti-p-FAK (1:5000; Abcam, Cambridge, MA, USA); polyclonal rabbitanti-ITGB2, anti-FAK (1:500; Wanleibio Co., Ltd., Beijing, China), monoclonal mouse anti-β-actin (1:1000; Beijing Zhongshan Golden Bridge Biotechnology Co., Ltd., Beijing, China). The membranes were washed with TBST three times and incubated with a secondary antibody (1:5000; Beijing Zhongshan Golden Bridge Biotechnology Co., Ltd., Beijing, China) for 1 h at 37 °C.

### 4.7. MTT Assay

Cell viability was detected by MTT (3-(4,5-dimethylthiazol-2-yl)-2,5-diphenyltetrazolium bromide) assay. A total of 4 × 103 cells were seeded into each well of 96-well plates in quintuplicate and cultured for indicated time. 10 μL of MTT (5 mg/mL; Sigma-Aldrich, St. Louis, MI, USA) was added into each well and then incubated for 4 h at 37 °C. Next, 150 μL of dimethyl sulfoxide (DMSO) was added to the 96-well plates. Finally, the absorbance was measured at 492 nm on a microplate reader. The overall experiments were repeated at least three times.

### 4.8. Flow Cytometry

Cells (1 × 106) from each group were harvested and washed thrice with cold PBS, followed by fixation with 70% cold ethanol at 4 °C overnight. After being washed in PBS, the cells were incubated with propidium iodide (PI; Sigma-Aldrich) and RNaseA for 30 min at room temperature. The cell cycle distribution was measured by a FACSVantage SE flow cytometer (Becton-Dickinson, San Jose, CA, USA).

### 4.9. Wound Healing Assay

After 6 h of transfection, or when the degree of fusion reached 95% or more, 10 μL of a small pipette tip was used to draw a line in the middle of a six-well plate. After washing with PBS, 2 mL of a double-free medium was added and photographed. This time was recorded as 0 h. After 48 h, photos were taken at the same position in each group of cells, and the distance between each group was calculated using Adobe Illustrator software at 0 h and 48 h, and the rate of scratch healing was calculated. Scratch healing rate = (0h width of scratch − 48 h width of scratch)/0 h width of scratch × 100%.

### 4.10. Transwell Chamber Assay

Transwell chambers (24-wellTranswell chambers, 8-μm pore size; Corning, Inc., Corning, NY, USA) were used for invasion assays. The transwell membrane was coated with 1:4 diluted Matrigel at advance. The cells after infection for 48 h were resuspended by serum-free media and cell number was adjusted to 10^5^/mL. 200 μL cell suspension was seeded into upper chambers. The lower chamber contained medium with 10% FBS. Following 24-h incubation, the cells that invaded the lower surface of the chamber were fixed with 4% paraformaldehyde, stained with 0.1% crystal violet, and counted from five random fields by bright field microscopy. Each experiment was repeated thrice.

### 4.11. Statistical Analysis

All the experiments were repeated three times and analyzed with GraphPad Prism (Prism 5.0, GraphPad Software, San Diego, CA, USA). The student’s *t*-test was used to evaluate the differences between two groups. The *p* < 0.05 was considered statistically significant. * *p* < 0.05, ** *p* < 0.01, *** *p* < 0.001.

## Figures and Tables

**Table 1:** Specific primer.

Gene	Sequence	Product Length (bp)
*itgb2-as1*	Forward: AGGAGATGGAACGAGGAAA Reverse: TTAGTGGTCTGCGAAGGTG	231
*itgb2*	Forward: TTCGGGTCCTTCGTGGACA Reverse: ACTGGTTGGAGTTGTTGGTCA	148
*β-actin*	Forward: CCACGAAACTACCTTCAACTCC Reverse: GTGATCTCCTTCTGCATCCTGT	132

**Table 2:** siRNA sequence.

siRNA	Sense Strand	Anti-Sense Strand
siNC	5′-UUCUCCGAACGUGUCACGUTT-3′	5′-ACGUGACACGUUCGGAGAATT-3′
siITGB2-AS1	5′-GCAGACCACUAAACCUCAUTT-3′	5′-AUGAGGUUUAGUGGUCUGCTT-3′

## References

[1] GBD2015 Mortality and Causes of Death Collaborators (2016). Global, regional, and national life expectancy, all-cause mortality, and cause-specific mortality for 249 causes of death, 1980–2015: A systematicanalysis for the global burden of disease study 2015. Lancet.

[2] Rossi S., Schinzari G., Basso M., Strippoli A., Dadduzio V., D’Argento E., Cassano A., Barone C. (2016). Maintenance hormonal and chemotherapy treatment in metastatic breast cancer: A systematic review. Future Oncol..

[3] Fitzmaurice C., Allen C., Barber R.M., Barregard L., Bhutta Z.A., Brenner H., Dicker D.J., Chimed-Orchir O., Dandona R., Dandona L. (2017). Global, regional, and national cancer incidence, mortality, years of life lost, years lived with disability, and disability-adjusted life-years for 32 cancer groups, 1990 to 2015: A systematic analysis for the global burden of disease study. JAMA Oncol..

[4] Fan L., Zheng Y., Yu K.D., Liu G.Y., Wu J., Lu J.S., Shen K.W., Shen Z.Z., Shao Z.M. (2009). Breast cancer in a transitional society over 18 years: Trends and present status in Shanghai, China. Breast Cancer Res. Treat..

[5] Chen C., Sun S., Yuan J.P., Wang Y.H., Cao T.Z., Zheng H.M., Jiang X.Q., Gong Y.P., Tu Y., Yao F. (2016). Characteristics of breast cancer in Central China, literature review and comparison with USA. Breast.

[6] Fan L., Strasser-Weippl K., Li J.-J., Louis J.S., Finkelstein D.M., Yu K., Chen W., Shao Z., Goss P.E. (2014). Breast cancer in China. Lancet Oncol..

[7] Quail D.F., Olson O.C., Bhardwaj P., Walsh L.A., Akkari L., Quick M.L., Chen I.C., Wendel N., Ben-Chetrit N., Walker J. (2017). Obesity alters the lung myeloid cell landscape to enhance breast cancer metastasis through IL5 and GM-CSF. Nat. Cell Biol..

[8] Iyengar N.M., Zhou X.K., Gucalp A., Morris P.G., Howe L.R., Giri D.D., Morrow M., Wang H., Pollak M., Jones L.W. (2016). Systemic correlates of white adipose tissue inflammation in early-stage breast cancer. Clin. Cancer Res. Off. J. Am. Assoc. Cancer Res..

[9] Pusztai L., Karn T., Safonov A., Abu-Khalaf M.M., Bianchini G. (2016). New strategies in breast cancer: immunotherapy. Clin. Cancer Res. Off. J. Am. Assoc. Cancer Res..

[10] Kang Q., Sun M.H., Cheng H., Peng Y., Montag A.G., Deyrup A.T., Jiang W., Luu H.H., Luo J., Szatkowski J.P. (2004). Characterization of the distinct orthotopic bone-forming activity of 14 BMPs using recombinant adenovirus-mediated gene delivery. Gene Ther..

[11] Wang J., Weng Y., Zhang M., Li Y., Fan M., Guo Y., Sun Y., Li W., Shi Q. (2016). BMP9 inhibits the growth and migration of lung adenocarcinoma A549 cells in a bone marrow stromal cells derived microenvironment through the MAPK/ERK and NF-κB pathways. Oncol. Rep..

[12] Yuan S.X., Wang D.X., Wu Q.X., Ren C.M., Li Y., Chen Q.Z., Zeng Y.H., Shao Y., Yang J.Q., Bai Y. (2016). BMP9/p38 MAPK is essential for the antiproliferative effect of resveratrol on human colon cancer. Oncol. Rep..

[13] Zhang J., Liang J.H., Huang J.G. (2016). Bone morphogenetic protein 9 facilitates osteocarcinoma cell apoptosis and inhibits in vivo tumor growth. Genet. Mol. Res..

[14] Garcíaálvaro M., Addante A., Roncero C., Fernández M., Fabregat I., Sánchez A., Herrera B. (2015). BMP9-induced survival effect in liver tumor cells requires p38MAPK activation. Int. J. Mol. Sci..

[15] Wang K., Feng H., Ren W., Sun X., Luo J., Tang M., Zhou L., Weng Y., He T.C., Zhang Y. (2011). BMP9 inhibits the proliferation and invasiveness of breast cancer cells MDA-MB-231. J. Cancer Res. Clin. Oncol..

[16] Ren W., Liu Y., Wan S., Fei C., Wang W., Chen Y., Zhang Z., Wang T., Wang J., Zhou L. (2014). BMP9 inhibits proliferation and metastasis of HER2-positive SK-BR-3 breast cancer cells through ERK1/2 and PI3K/AKT pathways. PLoS ONE.

[17] Liu B., Sun L., Liu Q., Gong C., Yao Y., Lv X., Lin L., Yao H., Su F., Li D. (2015). A cytoplasmic NF-κB interacting long noncoding RNA blocks IκB phosphorylation and suppresses breast cancer metastasis. Cancer Cell.

[18] Niknafs Y.S., Han S., Teng M., Speers C., Zhang C., Wilder-Romans K., Iyer M.K., Pitchiaya S., Malik R., Hosono Y. (2016). The lncRNA landscape of breast cancer reveals a role for DSCAM-AS1 in breast cancer progression. Nat. Commun..

[19] Cogill S.B., Wang L. (2014). Co-expression network analysis of human lncRNAs and cancer genes. Cancer Inform..

[20] cBioportal for Cancer Genomics. http://www.cbioportal.org/.

[21] Gao J., Aksoy B.A., Dogrusoz U., Dresdner G., Gross B., Sumer S.O., Sun Y., Jacobsen A., Sinha R., Larsson E. (2013). Integrative analysis of complex cancer genomics and clinical profiles using the cBioPortal. Sci. Signal..

[22] Cerami E., Gao J., Dogrusoz U., Gross B.E., Sumer S.O., Aksoy B.A., Jacobsen A., Byrne C.J., Heuer M.L., Larsson E. (2012). The cBio cancer genomics portal: An open platform for exploring multidimensional cancer genomics data. Cancer Discov..

[23] Tanric. http://ibl.mdanderson.org/tanric/_design/basic/index.html.

[24] Li J., Han L., Roebuck P., Diao L., Liu L., Yuan Y., Weinstein J.N., Liang H. (2015). TANRIC: An interactive open platform to explore the function of lncRNAs in cancer. Cancer Res..

[25] GEPIA (Gene Expression Profiling Interactive Analysis). http://gepia.cancer-pku.cn/.

[26] Tang Z., Li C., Kang B., Gao G., Li C., Zhang Z. (2017). GEPIA: A web server for cancer and normal gene expression profiling and interactive analyses. Nucleic Acids Res..

[27] Dent R., Trudeau M., Pritchard K.I., Hanna W.M., Kahn H.K., Sawka C.A., Lickley L.A., Rawlinson E., Sun P., Narod S.A. (2018). Triple-negative breast cancer: Clinical features and patterns of recurrence. Clin. Cancer Res..

[28] Vennin C., Spruyt N., Dahmani F., Julien S., Bertucci F., Finetti P., Chassat T., Bourette R.P., Le Bourhis X., Adriaenssens E. (2015). H19 non coding RNA-derived miR-675 enhances tumorigenesis and metastasis of breast cancer cells by downregulating c-Cbl and Cbl-b. Oncotarget.

[29] Li Z., Li Y., Li Y., Ren K., Li X., Han X., Wang J. (2017). Long non-coding RNA H19 promotes the proliferation and invasion of breast cancer through upregulating DNMT1 expression by sponging miR-152. J. Biochem. Mol. Toxicol..

[30] Gupta R.A., Shah N., Wang K.C., Kim J., Horlings H.M., Wong D.J., Tsai M.C., Hung T., Argani P., Rinn J.L. (2010). Long non-coding RNA HOTAIR reprograms chromatin state to promote cancer metastasis. Nature.

[31] Huang J., Zhou N., Watabe K., Lu Z., Wu F., Xu M., Mo Y.Y. (2014). Long non-coding RNA UCA1 promotes breast tumor growth by suppression of p27 (Kip1). Cell Death Dis..

[32] Liu M. (2018). Unpublished work.

[33] Tufarelli C., Stanley J.A.S., Garrick D., Sharpe J.A., Ayyub H., Wood W.G., Higgs D.R. (2003). Transcription of antisense RNA leading to gene silencing and methylation as a novel cause of human genetic disease. Nat. Genet..

[34] Yu W., Gius D., Onyango P., Muldoon-Jacobs K., Karp J., Feinberg A.P., Cui H. (2008). Epigenetic silencing of tumour suppressor gene p15 by its antisense RNA. Nature.

[35] Pandey R.R., Mondal T., Mohammad F., Enroth S., Redrup L., Komorowski J., Nagano T., Mancini-Dinardo D., Kanduri C. (2008). Kcnq1ot1 antisense noncoding RNA mediates lineage-specific transcriptional silencing through chromatin-level regulation. Mol. Cell.

